# Consecutive Serial Non-Contrast CT Scan-Based Deep Learning Model Facilitates the Prediction of Tumor Invasiveness of Ground-Glass Nodules

**DOI:** 10.3389/fonc.2021.725599

**Published:** 2021-09-10

**Authors:** Yao Xu, Yu Li, Hongkun Yin, Wen Tang, Guohua Fan

**Affiliations:** ^1^Department of Radiology, Second Affiliated Hospital of Soochow University, Suzhou, China; ^2^Department of Radiology, Dushuhu Public Hospital Affiliated of Soochow University, Suzhou, China; ^3^Department of Advanced Research, Infervision Medical Technology Co. Ltd, Beijing, China

**Keywords:** ground-glass nodules (GGNs), deep learning - artificial neural network (DL-ANN), computed tomography, follow-up, convolutional neural network

## Abstract

**Introduction:**

Tumors are continuously evolving biological systems which can be monitored by medical imaging. Previous studies only focus on single timepoint images, whether the performance could be further improved by using serial noncontrast CT imaging obtained during nodule follow-up management remains unclear. In this study, we evaluated DL model for predicting tumor invasiveness of GGNs through analyzing time series CT images

**Methods:**

A total of 168 pathologically confirmed GGN cases (48 noninvasive lesions and 120 invasive lesions) were retrospectively collected and randomly assigned to the development dataset (*n* = 123) and independent testing dataset (*n* = 45). All patients underwent consecutive noncontrast CT examinations, and the baseline CT and 3-month follow-up CT images were collected. The gross region of interest (ROI) patches containing only tumor region and the full ROI patches including both tumor and peritumor regions were cropped from CT images. A baseline model was built on the image features and demographic features. Four DL models were proposed: two single-DL model using gross ROI (model 1) or full ROI patches (model 3) from baseline CT images, and two serial-DL models using gross ROI (model 2) or full ROI patches (model 4) from consecutive CT images (baseline scan and 3-month follow-up scan). In addition, a combined model integrating serial full ROI patches and clinical information was also constructed. The performance of these predictive models was assessed with respect to discrimination and clinical usefulness.

**Results:**

The area under the curve (AUC) of the baseline model, models 1, 2, 3, and 4 were 0.562 [(95% confidence interval (C)], 0.406~0.710), 0.693 (95% CI, 0.538–0.822), 0.787 (95% CI, 0.639–0.895), 0.727 (95% CI, 0.573–0.849), and 0.811 (95% CI, 0.667–0.912) in the independent testing dataset, respectively. The results indicated that the peritumor region had potential to contribute to tumor invasiveness prediction, and the model performance was further improved by integrating imaging scans at multiple timepoints. Furthermore, the combined model showed best discrimination ability, with AUC, sensitivity, specificity, and accuracy achieving 0.831 (95% CI, 0.690–0.926), 86.7%, 73.3%, and 82.2%, respectively.

**Conclusion:**

The DL model integrating full ROIs from serial CT images shows improved predictive performance in differentiating noninvasive from invasive GGNs than the model using only baseline CT images, which could benefit the clinical management of GGNs.

## Introduction

Lung cancer is one of the most fatal cancers worldwide, and pulmonary nodules may represent early lung cancers. The National Lung Screening Trial and Dutch-Belgian Randomized Lung Cancer Screening Trial demonstrated that early lung cancer screening programs using low-dose computed tomography (CT) of the chest should be implemented globally (1). With the increasing use of CT screening for lung nodules—in particular, the extensive application of high-resolution CT (HRCT)—ground-glass nodules (GGNs) are observed increasingly often. As HRCT scans of the chest are gradually emerging as part of a routine physical examination, reasonable and necessary management of screening-detected and incidentally detected indeterminate pulmonary nodules is warranted.

Persistent lung GGNs may represent noninvasive or invasive adenocarcinoma. The prognosis of noninvasive lesions (atypical adenomatous hyperplasia (AAH) and adenocarcinoma *in situ* (AIS)) is quite different from that of invasive lesions (minimally invasive adenocarcinoma (MIA) and invasive adenocarcinoma (IAC)). Moreover, different pathological subtypes of GGNs vary with respect to the surgical approaches and clinical nodule management strategies required. In general, conservative nodule management is appropriate for noninvasive GGNs, whereas invasive GGNs are suitable for surgical resection. The overall survival rate after surgery for noninvasive GGN patients can be close to 100%, and there is a promising 5-year survival rate of 80%–95% in the case of invasive GGNs. However, they are characterized by very slow growth, so regular follow-up management and a wait-and-see policy are advocated by many experts (2). The Fleischner Society Guidelines for management of incidental subsolid nodules was published in 2017 and recommended follow-up intervals ranging from 3 months to several years (3). Guidelines for GGN management mainly include qualitative characteristics and patient history (e.g., smoking history and cancer history). Follow-up has a crucial role in clinical decision-making and assessment of surgical indication, and is increasingly recommended by thoracic and pulmonary guidance.

Most previous CT-based quantitative studies have used single screening images to estimate the invasiveness of GGNs on the basis of size, density, and mass volume (4, 5). However, there are known limitations to this approach owing to inter- and intraobserver variability in morphological features of GGNs. Nevertheless, in short-term follow-up, it is difficult to evaluate the invasion characteristics of GGNs based on morphological characteristics such as volume-doubling time and mass volume (6). In recent years, artificial intelligence has achieved great progress in the automatic quantitative image characterization of medical images; in particular, deep learning (DL) algorithms have proved to be versatile and efficient (1, 7). The clinical application of AI is currently extensive. The image-based AI can be used to distinguish the tissues of a COVID-19 patient and assessed the severity of their infection. Additionally, DL model can exhibit superior performance to that of general physicians and general orthopedic surgeons on shoulder radiographs in fracture datasets (8, 9). Owing to its favorable performance, DL has been widely used for the early detection, molecular subtype diagnosis, and prognosis prediction of GGNs. Several previous studies have reported the application of DL in predicting tumor invasiveness of GGNs. However, those studies only focused on the development of CT imaging biomarkers from a single timepoint, none of them had used serial CT images including the follow-up scans (10, 11). Since the evolution of tumor invasiveness is a dynamic biological progression with stem cell and vascular contributions, CT scan at a single time point might not capture the tumor phenotype completely (12–15). Incorporation of serial CT images from routine follow-up exanimations could be beneficial to track the phenotypic changes of GGNs and achieve more accurate diagnosis. There are few reports on using medical images from multiple time points for diagnosis (13, 16, 17). To the best of our knowledge, only two studies have investigated the use of serial CT images with DL algorithms for diagnosis of nodule malignancy or prognostic prediction in lung cancer patients (13, 18). Therefore, it is unclear whether the performance of DL models for predicting tumor invasiveness of GGNs could be further improved by using serial CT imaging.

In the current study, we aimed to analyze the characteristics of lesions based on combined baseline scans and follow-up scan images *via* artificial intelligence. We used DL methods— specifically, convolutional neural networks (CNNs) and recurrent neural networks (RNNs)—to predict early-stage lung adenocarcinoma presenting as GGNs by incorporating baseline and 3-month follow-up CT images. Our results have potential implications for the use of DL-based analysis of routine follow-up CT scans in patients with GGNs, as DL can be applied to predict tumor invasiveness noninvasively and is beneficial in precision medicine as well as clinical therapy.

## Methods

### Study Population

**Figure 1:**
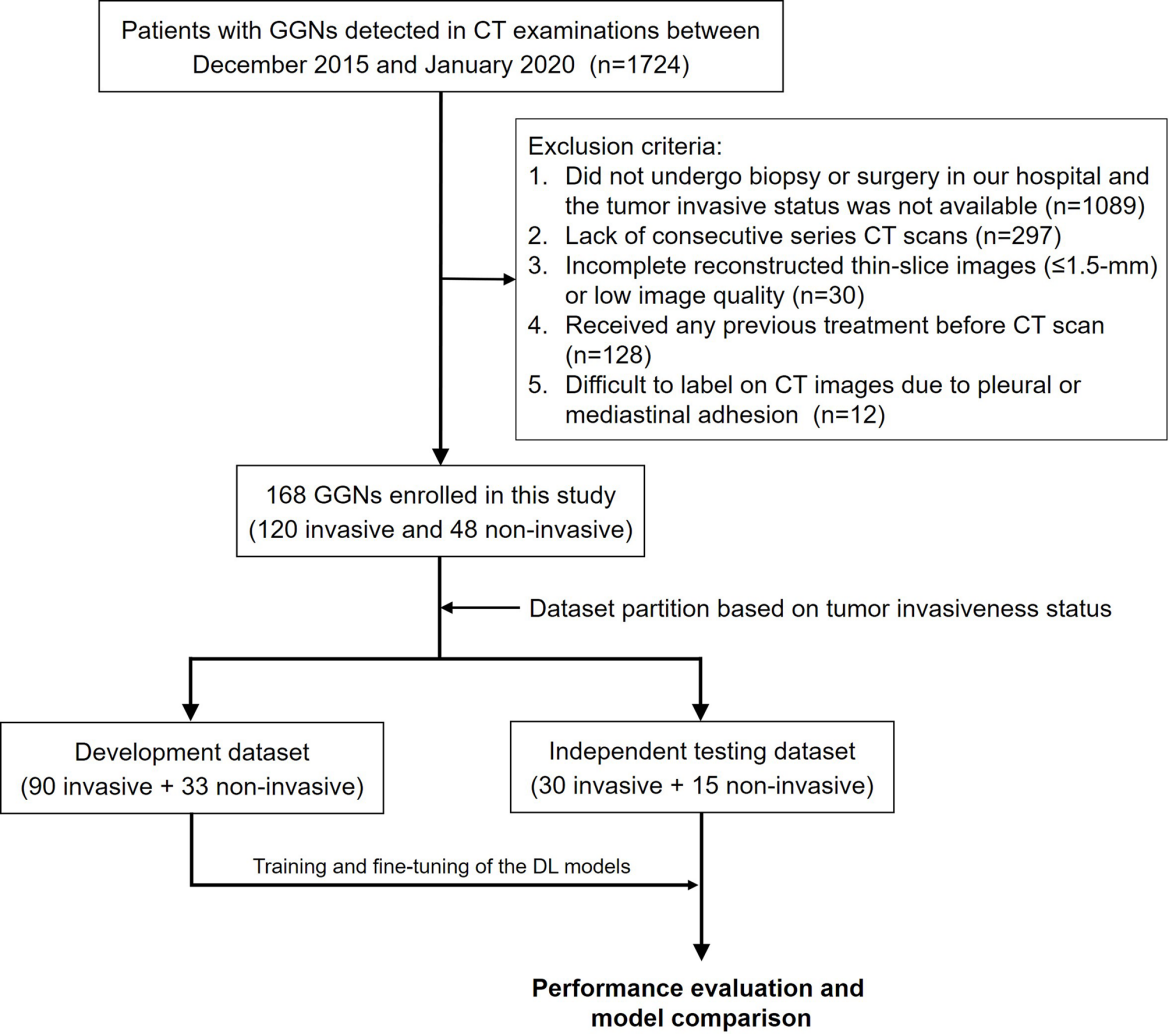
Patient enrollment and study design.

This study was approved by the institutional ethics committee of our hospital, and the informed consent requirement was waived. Data for 1724 patients who underwent CT examinations and were diagnosed as having GGNs between December 2015 and January 2020 were retrospectively retrieved from the picture archiving and communication system (PACS). The exclusion criteria were as follows: (1) the patient did not undergo biopsy or surgery in our hospital, and tumor invasiveness status was not available (n = 1089); (2) lack of consecutive series of CT scans (n = 297); (3) incomplete reconstructed thin-slice images (≤ 1.5 mm) or low image quality (n = 30); (4) patient had received any previous treatment before CT scan (n = 128); and (5) pleural or mediastinal adhesion was present and GGNs were difficult to label on CT images (n = 12). Finally, a total of 168 patients with 168 GGNs [13 atypical adenomatous hyperplasia (AAH), 107 minimally invasive adenocarcinoma (MIA), 35 adenocarcinoma *in situ* (AIS), and 13 invasive adenocarcinoma (IAC)] were enrolled, and two consecutive CT scans within about 3 months (82–109 days, median 93 days) for each patient were used in this study. The invasiveness of GGNs was later confirmed through pathological analysis. AAH and AIS were classified as noninvasive lesions, and MIA and IAC were classified as invasive lesions. The study workflow is depicted in **Figure 1**.

### CT Image Acquisition

All patients underwent CT scanning at our hospital with the 750 HD CT (Discovery, GE Healthcare, North Richland Hills, TX, USA) scanner or the 256 multidetector row scanner (Brilliance iCT, Philips Healthcare, Cleveland, OH, USA). Scan parameters were as follows: 0.625-mm slice thickness and 1.25-mm slice spacing; 120 kV voltage; automatic tube-current modulation with a mean tube current of 100 mA; 5 mm collimator, and a 512 × 512 matrix. All the thin-slice CT images were reviewed by a thoracic radiologist with more than 10 years of experience in chest CT for image qualitative evaluation.

### Annotation and Pretreatment of Tumor Regions

The GGNs in CT images were manually labeled by a radiologist with 5 years of experiences in chest imaging using the ITK-SNAP software (version 3.8.0, http://www.itksnap.org). The GGNs were segmented at the lung window set (Window Width 1500 Hu, Window Level -450 Hu) by carefully drawing a region of interest (ROI) that traced the edge of the GGN on all axial images until the entire GGN was covered. The annotation results were further checked by another senior radiologist with 10 years of experience. When the boundary of the GGN was uncertain, an expert radiologist with more than 20 years of experience in lung cancer diagnosis was consulted for the final decision. All radiologists were blinded to the pathological results.

**Figure 2:**
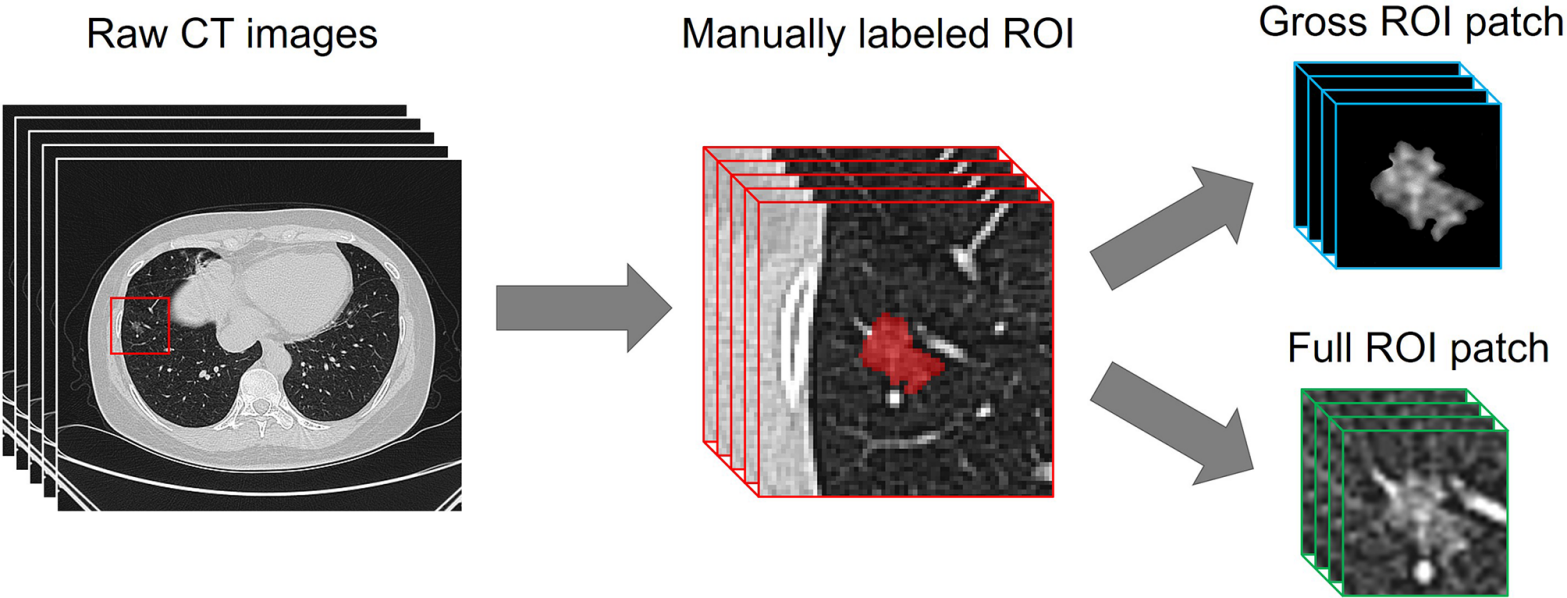
Examples of the automatedly generated gross ROI patch and full ROI patch.

Before the development of the DL models, a resampling approach was used for data pretreatment. The CT images were rescaled to a uniform size with 1-mm isotropic voxel spacing, then each manually labeled ROI was transformed and defined as follows. (i) A 64 × 64 × 64-pixel three-dimensional (3D) patch containing the nodule region which was cropped from each CT image, and the size was determined based on the largest ROI. (ii) The pathologically identified label of tumor invasiveness. As previous studies have indicated that the peritumoral region provides valuable insight for determining the prognosis of lung cancer (19, 20), both the gross ROI patch and the full ROI patch containing perinodular regions were automatically generated, as the nonlesion area of the 3D patch was left padding zero or reserving perinodular imaging (**Figure 2**).

### Development of the Baseline Model

A baseline model was constructed by using the image and demographic features including tumor size, location, age, gender, cancer history, and smoking history. The logistic regression (LR) analysis was used as the classifier. The baseline model was built in the development dataset and validated in the independent testing dataset.

### Construction of the DL Models

Fifteen noninvasive lesions and 30 invasive lesions were firstly randomly selected to serve as the independent testing dataset, and the remaining samples (33 noninvasive and 90 invasive) were used as the development dataset. Owing to the limited amount of training data, data augmentation techniques including flipping (perpendicular to the *x*- and *y*-axis), random shifting (15% towards the eight vertexes of the 3D patch), random rotation (90°, 180°, and 270° perpendicular to the *z*-axis), mirroring, and random brightness contrast (80%, 90%, 110%, and 120%) were used in the development of the neural networks. After data augmentation, the sample size increased to 19 times that of the original, yielding a total of 2,337 samples in the development dataset.

**Figure 3:**
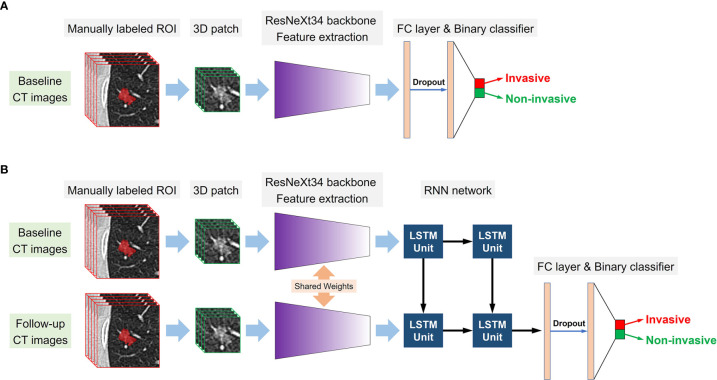
Conceptual architecture of the single-DL model using only baseline CT images **(A)** and the serial-DL model integrating serial CT images at multiple timepoints **(B)**.

We employed a modified 3D ResNeXt34 as the backbone network of the DL models, as the 3 × 3 2D convolution filters were replaced by 3 × 3 × 3 3D convolution filters (21). Transfer learning approach was applied to improve the robustness and generalization of the DL models (22). To pretrain the modified 3D ResNeXt34 network in this study, a total of 178 pulmonary nodules were manually labeled on the TCGA-LUAD, CPTAC-LUAD, TCGA-LUSC, and CPTAC-LSCC datasets which were downloaded from The Cancer Imaging Archive (TCIA) database. For the prediction of tumor invasiveness based on single or serial CT images, two kinds of DL models were designed: the single-DL model using only the baseline CT images as inputs and the serial-DL model using two consecutive series (baseline and follow-up) CT images as inputs. For the single-DL model, the 3D ResNeXt34-based CNN with a fully connected layer was used to extract high-dimensional features from the imaging data, followed by a soft-max output layer to predict the probability of tumor invasiveness. The neural architecture for the serial-DL model included ResNeXt34-based CNN merged with a RNN (**Figure 3A**). In brief, two weight-sharing 3D ResNeXt34 networks were used for feature extraction from two consecutive series CT images, and the outputs of each CNN model were fed into the RNN with a long short-term memory (LSTM) architecture as time-varying inputs (**Figure 3B**). Therefore, four DL models were designed in our study: a single-DL model using gross ROI patches as inputs (model 1), a serial-DL model using gross ROI patches as inputs (model 2), a single-DL model using full ROI patches as inputs (model 3), and a serial-DL model using full ROI patches as inputs (model 4).

**Figure 4:**
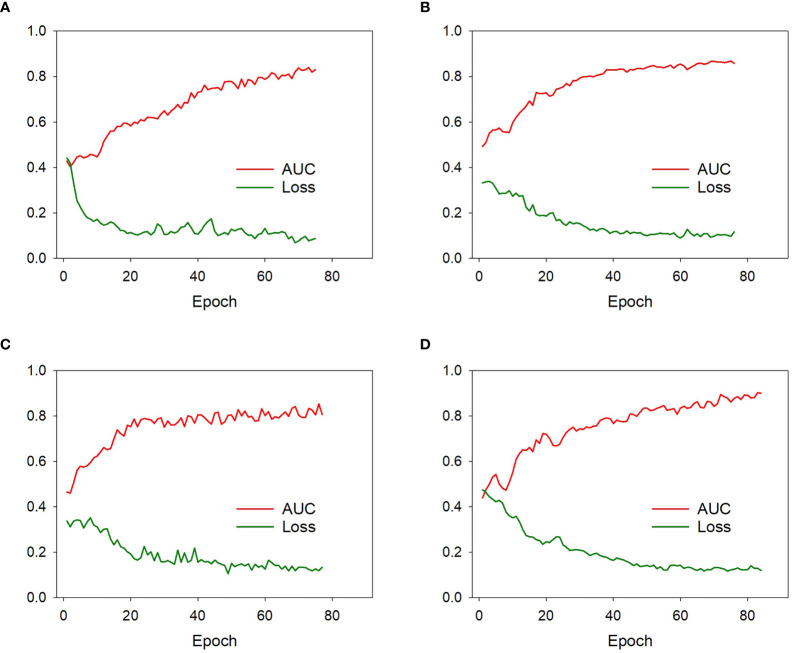
The model efficiency (AUC) and cross-entropy loss function corresponding to each epoch of the model 1 **(A)**, model 2 **(B)**, model 3 **(C)**, and model 4 **(D)** during training process. The model efficiency corresponding to each epoch gradually increased while the model loss function decreased and eventually stabilized.

The proposed single-DL models were trained based on the binary cross-entropy loss function; the weights of hidden layers were randomly initialized by Xavier, and the initial learning rate was set to 0.0001. Adam was used as the optimizer in the training stage owing to its fast convergence and weight-dependent learning rate, and the beta 1 and beta 2 parameters were 0.9 and 0.99, respectively. In addition, a weighted oversampling technique was used to train the model; only resampled minibatches with a noninvasive/invasive ratio of 1:1 was selected for training. The minibatch size was 24, and the dropout rate was set to 0.5; other parameters were set to their default values. As for the serial-DL models, there were three cells in the LSTM unit with the dropout rate set to 0.8, and each cell contained 512 features. The training was stopped when the loss of function was stable (23). Since deep learning models based on small sample size could be subject to obvious overfitting after a certain number of epochs, the early stopping method was used to halt parameter iteration for the model. The number of modeling epochs was set between 60 and 100 in this study. The changes in model efficiency (AUC) and cross-entropy loss function index corresponding to each epoch in the training process of the DL models were shown in **Figure 4**.

The code of these DL models was open sourced at https://github.com/TangWen920812/3d-resnext-lstm.

Supervised training was performed on a computer with a Core i7-6700 K central processing unit (Intel, Santa Clara, CA, USA), 32 GB memory, and a GeForce GTX 1080 graphics processing unit (NVIDIA, Santa Clara, CA, USA). Python 3.6.8 (https://www.python.org) and the Mxnet 1.5.0 (https://mxnet.incubator.apache.org) framework for neural networks were used to construct the DL models. The development and independent of the DL models were performed with InferScholar platform version 3.3 (InferVision, China).

### Development of the Combined Model

To integrate both serial CT images and clinical information, a combined model was constructed by incorporating the following candidate variables: age, gender, GGN size, GGN location, cancer history, smoking history, and the invasiveness probability calculated by the serial-DL model. The combined model was developed in the development dataset by using linear support vector machine (linear SVM) classifier, and the prediction value was calculated using following formula:


$$\begin{array}{c} \text{Prediction value}=-0.4061*\text{gender}\;\left(\text{male}=1,\text{ female}=-1\right)\\ -0.0316*\text{age}+0.5521*\text{GGN}\;\text{size}+0.6422*\text{RUL location }\left(\text{yes}=1,\;no=0\right)\\ -0.5790*\text{RML location }\;\left(\text{yes}=1,\text{ no}=0\right)-1.9971*\text{RLL location }\left(\text{yes}=1,\text{ no}=0\right)\\ +1.1625*\text{LUL location }\left(\text{yes}=1,\text{ no}=0\right)+0.7715*\text{LLL location }\left(\text{yes}=1,\text{ n}o=0\right)\\ +1.0314*\text{cancer history }\left(\text{yes}=1,\text{ no}=0\right)+0.4313*\text{smoking history }\left(\text{yes}=1,\text{ n}=0\right)\\ +39.818*\text{invasiveness probability}-14.6215\text{ (LUL left upper lobe};\text{ LLL},\;\text{left lower lobe};\text{ RUL},\\ \text{ right upper lobe};\text{ RML},\text{ right middle lobe};\text{ RLL},\text{ right lower lobe}) \end{array}$$

In addition, the decision curve analysis (DCA) was applied to assess the clinical usefulness of the combined model as well as the deep learning models on the independent testing dataset.

### Statistical Analysis

In order to evaluate the performance of the DL models for the discrimination of noninvasive from invasive lesions, a receiver operating characteristic (ROC) curve was plotted for the calculation of sensitivity and specificity, and the area under the curve (AUC) was determined. The sensitivity, specificity, and accuracy were calculated under optimal threshold according to the maximum Youden index (24). Delong’s test was used to compare the differences between two or more AUCs of different models. The association between categorical variables was assessed by Chi-square test or Fisher’s exact test, and the Mann–Whitney *U* test was performed to evaluate the differences among variables with a continuous distribution. The DCA curve was plotted using the “rmda” package. All analyses were performed using Prism 5 for Windows (version 5.01), and a two-sided *p*-value <0.05 was considered statistically significant.

## Results

### Patient Characteristics

The clinicopathologic characteristics of the enrolled patients in the independent and datasets are summarized in **Table 1**. There were no significant differences in gender, age, cancer history, or smoking history between patients with noninvasive nodules and those with invasive nodules in either the development or the independent testing dataset (all *p* > 0.05). The prevalence of GGN showed a greater tendency to occur in the upper lobe in the noninvasive group compared with the invasive group (70.8% [34/48] in the noninvasive group *vs.* 47.5% [57/120] in the invasive group, *p* = 0.03). The average GGN in the invasive group was larger than that of the noninvasive group across all patients (*p* = 0.02); however, the differences were not significant in the development dataset (*p* = 0.08) or the independent testing dataset (*p* = 0.10).

**Table 1:** The clinicopathologic characteristics of enrolled patients.

	All patients			Development dataset			Independent dataset		
	Noninvasive	Invasive	*p*-Value	Noninvasive	Invasive	*p*-Value	Noninvasive	Invasive	*p*-Value
Gender			0.67			0.19			0.27
Male	14	39		7	30		7	9	
Female	34	81		26	60		8	21	
Age (years)			0.16			0.11			0.83
Mean	46.8	49.8		45.7	49.7		49.1	50.0	
SD	10.4	13.0		11.3	12.4		8.0	14.8	
GGN size (mm)			0.02			0.08			0.10
Mean	7.6	9.1		7.8	9.0		7.3	9.3	
SD	2.3	3.9		2.4	3.8		1.9	4.3	
GGN location			0.06			0.05			0.53
LUL	18	30		11	23		7	7	
LLL	3	19		2	13		1	6	
RUL	16	27		13	20		3	7	
RML	2	17		0	13		2	4	
RLL	9	27		7	21		2	6	
Cancer history			0.44			0.52			0.71
Yes	4	15		3	12		1	3	
No	44	105		30	78		14	27	
Smoking history			0.69			0.80			0.36
Yes	13	29		7	21		6	8	
No	35	91		26	69		9	22	

GGN, ground-glass nodules; LUL, left upper lobe; LLL, left lower lobe; RUL, right upper lobe; RML, right middle lobe; RLL, right lower lobe.

### Performance of Different Predictive Models in the Independent Testing Dataset

In the independent testing dataset, the baseline model showed limited discrimination capability with the AUC, accuracy, sensitivity, and specificity achieving 0.562 (95% CI, 0.406–0.710), 64.4%, 67.7%, and 60.0%, respectively. The result indicated that an effective diagnosis of tumor invasion in GGNs was not possible when only using the clinical variables.

**Figure 5:**
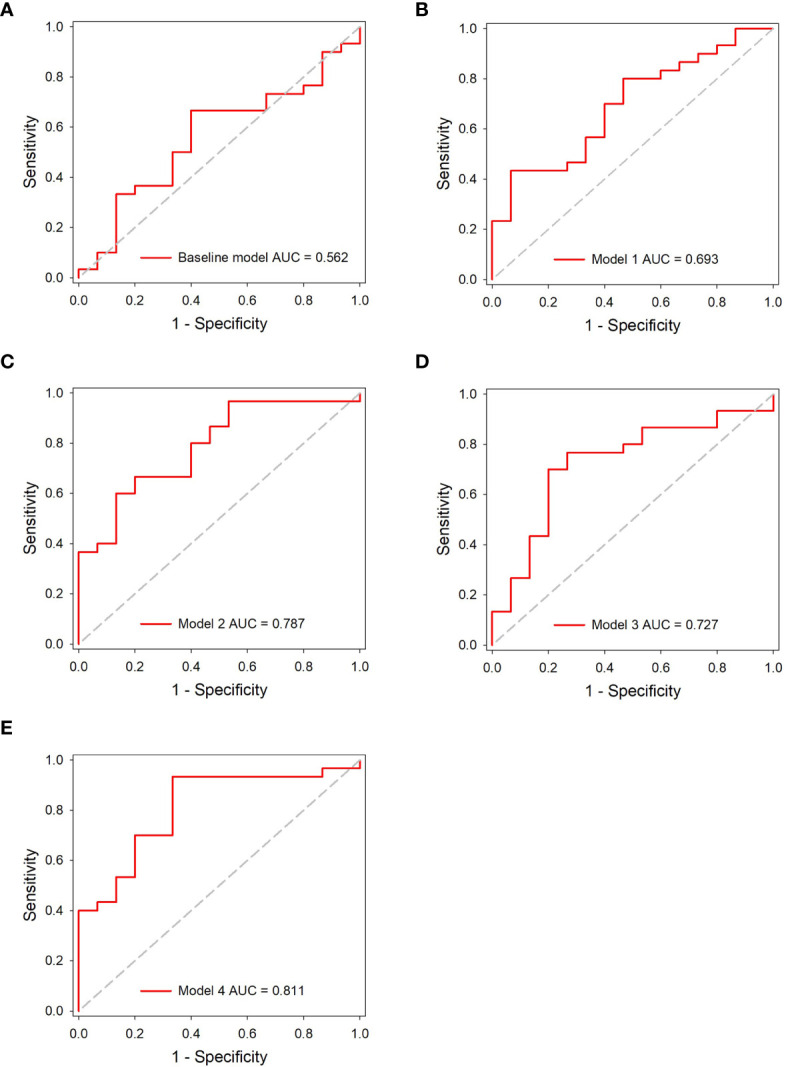
ROC analysis of the predictive models in the independent testing dataset. **(A)** The baseline model. **(B–E)** The DL models.

The accuracy of DL models was 66.6% (model 1), 71.1% (model 2), 75.6% (model 3), and 84.4% (model 4) in the independent testing dataset. The AUCs of models 1 and 2 (using gross ROI patches as inputs) were 0.693 (95% CI, 0.538–0.822) and 0.787 (95% CI, 0.639–0.895), respectively (**Figures 5A, B**), whereas that of models 3 and 4 (using full ROI patches as inputs) were 0.727 (95% CI, 0.573–0.849) and 0.811 (95% CI, 0.667–0.912), respectively (**Figures 5C, D**). The serial-DL models and the combined model outperformed the baseline model with significant differences (Delong’s test, *p* = 0.046, 0.022, and 0.024 for model 2, model 4, and combined model, respectively). The full ROI patch-based DL models showed an increased performance tendency than the gross ROI patch-based models; however, the differences were not statistically significant (model 3 *vs.* model 1, *p* = 0.753, model 4 *vs.* model 2, *p* = 0.796). In addition, the AUC of serial CT image-based DL models was also higher than that of single CT image-based DL models (model 2 *vs.* model 1, *p* = 0.187, model 4 *vs.* model 3, *p* = 0.383). The accuracy of the serial-DL model using full ROI was significantly higher than that of the single-DL model using gross ROI (model 4 *vs.* model 1, 84.4% *vs.* 66.6%, Chi-square test *p* = 0.049), indicating that the spatial pattern of perinodular regions and incorporation of serial CT images could facilitate the prediction of tumor invasiveness in patients with GGNs.

### Evaluation of the Combined Model

**Figure 6:**
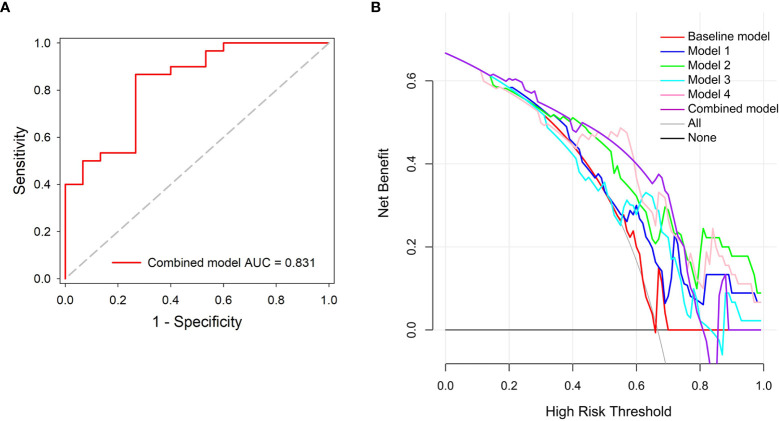
Performance evaluation of the combined model. **(A)** ROC analysis. **(B)** Decision curve analysis for the predictive models; the combined model had higher net benefit compared with the other models across majority range of threshold probabilities.

The combined model showed the best performance with AUC, sensitivity, specificity, and accuracy achieving 0.831 (95% CI, 0.690–0.926), 86.7%, 73.3%, and 82.2%, in the independent testing dataset, respectively (**Figure 6A**). The details of the predictive performance of the baseline model, the DL models, and the combined model are summarized in **Table 2**. DCA was used to evaluate the clinical usefulness of the different predictive models. The results showed that the combined model had a slightly higher overall net benefit compared with the DL models across the majority of probability threshold (**Figure 6B**).

**Table 2:** Performance comparison of the predictive models in the independent dataset.

Models	AUC (95% CI)	p-Value	Cut-off threshold	Accuracy	Sensitivity	Specificity
Baseline	0.562 (0.406~0.710)	Reference	0.5396	64.4% (29/45)	66.7% (20/30)	60.0% (9/15)
Model 1	0.693 (0.538~0.822)	0.314	0.5239	66.7% (30/45)	70.0% (21/30)	60.0% (9/15)
Model 2	0.787 (0.639~0.895)	0.046	0.5248	71.1% (32/45)	66.7% (20/30)	80.0% (12/15)
Model 3	0.727 (0.573~0.849)	0.197	0.4918	75.6% (34/45)	76.7% (23/30)	73.3% (11/15)
Model 4	0.811 (0.667~0.912)	0.022	0.4685	84.4% (38/45)	93.3% (28/30)	66.7% (10/15)
Combined	0.831 (0.690~0.926)	0.024	0.6570	82.2% (37/45)	86.7% (26/30)	73.3% (11/15)

AUC, area under the curve.

## Discussion

In this study, we developed novel DL models to detect the invasiveness of GGNs based on consecutive CT thin-scanned images (baseline and 3-month follow-up scans). We found that the peritumoral region could contribute to invasiveness prediction. Notably, integrating consecutive serial CT images further improved the performance of DL models for predicting tumor invasiveness of GGNs. In addition, the combination of clinical variables and risk probability calculated by DL model showed favorable capability in distinguishing noninvasive GGNs from invasive GGNs.

Previous studies have reported that the size of GGNs is a critical risk factor for potential invasiveness (3–5, 21). Lee et al. found that the optimal cutoff size for preinvasive lesions was less than 10 mm (sensitivity, 53.33%; specificity, 100%) in a pure GGN cohort; this could be used as a selection criterion to identify patients suitable for sublobar resection (25). In addition, Kim showed that 8 mm was the optimal cutoff value for discrimination of noninvasive GGNs from invasive GGNs (26). In short, these results combined with those of previous studies indicate that the clinical feature of size is indeed highly correlated with the invasiveness of GGNs (27). Notably, we also observed significant differences in size between the preinvasive cohort and the invasive cohort in the all-patient dataset (*p* < 0.05). However, there were no such significant differences in either the independent testing dataset or the development dataset, although the average GGN size of the invasive group was larger than that of the noninvasive group in all patients.

The largest lung cancer screening trial in Europe showed that malignant tumors were localized predominantly in the periphery of lungs and the right upper lobe (27). Interestingly, in our study, the GGNs in the noninvasive group showed a greater tendency to occur in the upper lobe compared with the invasive group (noninvasive group 34/48 *vs.* invasive group 57/120). This bias is probably introduced in our study because of the difference between tumor malignancy and tumor invasiveness.

However, previous studies had certain limitations. First, most studies only considered whether clinical characteristics such as smoking *vs.* nonsmoking or tumor history (yes *vs.* no) were related to GGN growth. Few studies have evaluated the weights of CT images and clinical information for predicting GGN invasiveness with specific numerical formulas, which could be better verified and recognized by radiologists. Second, the most of reported DL algorithms were applied to lung nodule classification as benign or malignant, and they focus on a single scan for the model input.

Most of the previous studies only considered the relation between the quantitative radiographic characteristic and pathologic classification that are limited at the single time-point (19, 21, 28). In our study, we applied different DL algorithms to predict the invasiveness of GGNs. The single-DL models and serial-DL models that are based on whether they used single or serial CT images were proposed, and their performance was compared. This is a different approach from the current method of predicting malignant nodules based on a single CT scan (26, 28). We combined baseline and 3-month follow-up continuous CT scan images to obtain the original features and changed features that maximized the risk of tumor invasion. Recently, numerous studies and guidelines have advocated that early follow-up of patients with GGN should replace unnecessary surgical resection (3, 5, 19). However, subtle changes between the short-term follow-up images and the baseline CT images are often invisible to mostly radiologists, hence the need to evaluate invasiveness using DL algorithms. Previous studies have shown that an increase of 2 mm or more in diameter indicates that a GGN is growing; this change is often related to the malignant characteristics of the nodule (26, 29). Qi et al. showed that compared with the 2D diameter, a 20% increase in volume can reflect the growth of GGNs with greater sensitivity and accuracy (30). In addition, the development of solid proportions is considered strong evidence for clinical management of part-solid nodules (31). Recently, increasing numbers of studies have shown that high-throughput extraction of details that are not obvious or visible to the human eye, using radiomics and artificial intelligence, has great advantages and promising applications (11, 12, 32–34). Therefore, considering the results of the above studies, it may be reasonable to infer that the DL model incorporating both follow-up and baseline CT scans could better predict the invasiveness of nodules, enabling GGNs to be managed more rationally and avoiding unnecessary surgical resection.

Several studies have reported that radiomics and DL algorithms could be used to detect the invasiveness of GGNs. However, most of these studies just focused on the nodules themselves, few had investigated the contribution of the microgrowth environment to the prediction of tumor invasiveness. Wu suggested that there were differences between ICA and MIA/AIS in the radiomic features of cluster prominence and the gray level run-length matrix in the surrounding area of the tumor (35). Wang and Beig et al. found that clinical interpretation of peritumoral radiomics features could be used to differentiate adenocarcinoma from granuloma, predict the characteristics of lymph node metastasis, and evaluate recurrence rates after surgery (36, 37). Those reports indicated that the peritumor regions of GGNs could be used for diagnosis of invasiveness.

In our study, the proposed DL algorithms could be categorized into two groups (gross ROI-DL and full ROI-DL) depending on the size of the extracted GGN ROI range. The full ROI patch containing both the nodules and perinodular regions provided information on the nodules themselves as well as their microgrowth environment. As expected, the full ROI group (models 3 and 4) achieved higher AUC values for predicting tumor invasiveness than the gross ROI group (models 1 and 2). These findings suggested that the spatial pattern of perinodular regions could also have a role in tumor invasiveness prediction. In addition, the results were similar to those of previous radiomics studies, in which combining texture features extracted from both intranodular and perinodular regions led to better performance compared with the single intranodular-based approach (35, 37). Unfortunately, there are currently no relevant authoritative studies or guidance on the specific size of the perinodular region (38). Furthermore, the combined model that integrated the serial CT images and clinical information involved calculations using a specific formula for prediction. DCA demonstrated that the combined model had a moderate increase in overall net benefit compared with the DL models (models 1–4).

To our knowledge, this is the first study combining the serial CT imaging to corroborate the quantitative predictive relationship between clinical-radiological characteristics and invasiveness. Most previous studies focused on the time-to-growth characteristics of tumors or the effectiveness of computer-aided diagnosis. Yoshihisa reported that smoking history and initial lesion diameter were strongly related to GGN growth (18). Matsuguma et al. analyzed the growth of 174 subsolid GGNs during the follow-up period and found that history of lung cancer was a significant predictive factor in GGN growth (39). In recent years, various DL models have been widely used to evaluate and detect changes in GGNs and have shown excellent performance compared with radiologists (40, 41). Zhao et al. reported a DL system based on 3D CNNs, and multitask learning, which achieved better classification performance than senior and junior doctors in pathological labeling of GGNs (41). Moreover, Ding et al. applied two models for distinguishing degree of nodule invasiveness, the lung DL model and dense model; both modes showed high performance in terms of AUC (0.88 and 0.86, respectively), especially the lung DL model (42).

Our study also had several limitations. First, it was a retrospective single-center study and the number of GGNs used for model development was limited; a prospective multicenter study with a larger sample size will be required in the future. Second, the ROIs were mainly manually segmented. Automatic detection and segmentation of GGNs will be considered in our future research. Third, whether incorporating more time-point serial CT images (e.g., 6-month follow-up and 12-month follow-up) could further benefit DL models in predicting tumor invasiveness still needs investigation.

In conclusion, integration of consecutive serial CT images improves the predictive efficacy of DL models in differentiating noninvasive GGNs from invasive GGNs, and the performance could be further improved by incorporating clinical information. The proposed DL models in this study show favorable performance and might have the potential to assist clinicians in tailoring precise therapy.

## Data Availability Statement

The raw data supporting the conclusions of this article will be made available by the authors, without undue reservation.

## Ethics Statement

This study was approved by the Institutional Ethics Committee of the Second Affiliated Hospital of Soochow University, and the informed consent requirement was waived.

## Author Contributions

HY and WT performed the deep learning model, analyzed the data. All authors contributed to the article and approved the submitted version.

## Conflict of Interest

HY and WT were employed by Beijing Infervision Technology Co., Ltd.

The remaining authors declare that the research was conducted in the absence of any commercial or financial relationships that could be construed as a potential conflict of interest.

## Publisher’s Note

All claims expressed in this article are solely those of the authors and do not necessarily represent those of their affiliated organizations, or those of the publisher, the editors and the reviewers. Any product that may be evaluated in this article, or claim that may be made by its manufacturer, is not guaranteed or endorsed by the publisher.
